# Age–Treatment Interactions in Out-of-Hospital Cardiac Arrest: A Nationwide Registry Analysis

**DOI:** 10.3390/jcm15020705

**Published:** 2026-01-15

**Authors:** Boldizsár Kiss, Ádám Pál-Jakab, Bettina Nagy, Gábor Koós, Gábor Csató, György Pápai, Béla Merkely, Endre Zima

**Affiliations:** 1 Heart and Vascular Centre, Semmelweis University, H-1122 Budapest, Hungary; kiss.boldizsar@semmelweis.hu (B.K.);; 2 Hungarian National Ambulance Service, H-1055 Budapest, Hungary; 3 Hungarian Resuscitation Council, H-1011 Budapest, Hungary; 4 Institute of Anesthesiology and Perioperative Care, Semmelweis University, H-1082 Budapest, Hungary

**Keywords:** out-of-hospital cardiac arrest, elderly, age, prediction, resuscitation

## Abstract

**Introduction**: Population aging in Europe is ongoing and linked to poorer outcomes after out-of-hospital cardiac arrest (OHCA), yet age alone should not guide treatment. We aimed to describe age-related survival, identify independent predictors, and develop a predictive model using EMS data. **Methods**: We analyzed 147,962 adult OHCA cases from the Hungarian National EMS registry. Variables included initial rhythm, witness status, location, and sex. The primary outcome was survival to hospital admission. Multivariable logistic regression assessed independent predictors and age × treatment interactions; performance was evaluated with AUC, Brier score, and cross-validation. **Results**: Overall survival was 8.8%; elderly patients had lower survival (7.3%) than non-elderly (11.7%, *p* < 0.001). VF/VT (adjusted OR 5.34), medical personnel witness (OR 4.52), and AED shock (OR 3.52) were the strongest predictors. Age attenuated the survival benefit of VF/VT (interaction OR 0.914) and the protective effect of female sex (interaction OR 0.882; both *p* < 0.001). Model performance was good (AUC 0.784; Brier 0.0705). **Conclusions**: Age independently predicts survival after OHCA, but substantial treatment benefits persist in the elderly. Age–treatment interactions support geriatric-tailored resuscitation strategies and potential integration of this high-performing model into clinical decision support systems.

## 1. Introduction

Aging of the European population is a long-term process which affects all the countries in the European Union (EU). The actual EU’s population structure (according to Eurostat data) shows the signs of not only the aging, but the progressive aging of the older population as well. Between 2019 and 2050, the elderly population (aged 65 years or more) in the EU will increase significantly, rising from 90.5 million to reach 129.8 million [1,2].

Age is a significant factor influencing both the incidence and outcomes of cardiac arrest. The risk of cardiac arrest increases with age. Elderly patients accounted for more than 80% of all out-of-hospital cardiac arrest (OHCA) cases [3,4]. Based on epidemiology data, the proportion of elderly OHCA patient population will increase higher in the near future [5].

Older age is consistently associated with lower survival rate and poorer neurological outcome after OHCA. Younger patients have higher rates of return of spontaneous circulation (ROSC), survival to hospital discharge, and good neurological outcomes compared to older patients. Survival after OHCA drops from 16.7% (under 20 years) to 1.7% (aged 95–99), with a similar trend for good neurological outcomes [6,7,8].

In everyday practice, without knowing the patients’ medical history and the circumstances of sudden cardiac death, one may decide to stop resuscitation or to do the intensity of any post-resuscitation intensive care efforts based on the patient’s age only. Older patients often receive less intensive resuscitation and post-arrest care, which may contribute to lower survival rates [9,10]. Age alone should not be the sole criterion for treatment decisions. While outcomes worsen with age, there is no specific age cutoff at which recovery becomes impossible. By the knowledge from the literature so far some elderly patients, especially those with favorable initial rhythms, still achieve meaningful recovery [11,12,13].

Even papers and guidelines do not support decision-making based on only a single factor such as age. As some elderly survivors achieve good outcomes, this kind of decision is not ethically justifiable [7,14].

Multiple comorbidities are generally associated with poorer survival and neurological recovery after cardiac arrest. However, the quality of resuscitation can play an even more critical role, and, sometimes, outweigh the negative impact of comorbidities on neurological outcomes [15,16,17].

Outcomes after OHCA remain poor and are driven largely by prehospital determinants of ROSC and survival to hospital admission. During the first COVID-19 wave, an individual patient data meta-analysis reported more arrests occurring at home, fewer shockable rhythms, reduced ratio of bystander CPR in high-burden regions, and an independent decrease in survival to hospital admission (with higher risk of prehospital death) [18].

The impact of adrenaline in prehospital resuscitation also remains contentious, as higher cumulative doses—often a surrogate of prolonged resuscitation—have been associated with a higher admission rate but same or worse mortality [19].

Using the Hungarian National Ambulance Service (HNAS) registry, we aimed to quantify age-stratified survival to hospital admission (and ROSC) among adult (≥18 years) out-of-hospital cardiac arrest patients in whom EMS initiated or continued resuscitation. We further sought to identify independent prehospital predictors (including age × treatment effect modification) and to develop and internally validate an EMS-based prognostic model for early risk stratification.

## 2. Materials and Methods

### 2.1. Study Design and Setting

We conducted a retrospective cohort study using the Hungarian National Ambulance Service (Budapest, Hungary) registry, analyzing all OHCA cases from 1 November 2018 to 28 February 2025. Hungary’s centralized HNAS system provides comprehensive coverage with standardized protocols and mandatory reporting, ensuring a low rate of data loss. The study was approved by the National Ethics Committee of Hungary (Budapest, Hungary). Ethical approval was granted for the collection and analysis of data related to OHCA cases, integral to our observational assessment of OHCA epidemiology, regional variations, and mortality rates in Hungary. The study was assigned ethical approval numbers (Reference Numbers: IV/3043/2021/EKU and IV/3043-3/2021/EKU). All procedures and data handling adhered strictly to established ethical standards and regulations and were conducted in accordance with the Declaration of Helsinki [20].

### 2.2. Study Population and Data Selection

**Figure 1:**
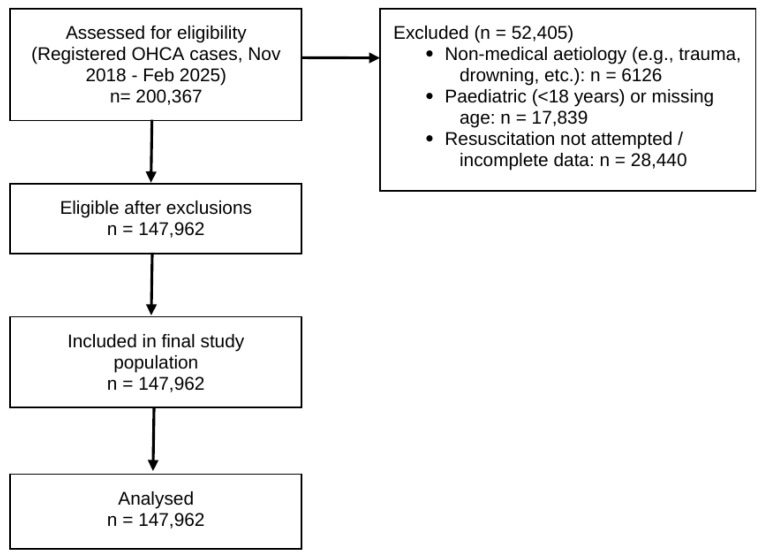
Flowchart of study population selection.

During the study period from 1 November 2018 to 28 February 2025, the Hungarian National Ambulance Service registered a total of 200,367 cases. From this initial dataset, we excluded 6126 cases due to non-medical etiologies (including trauma, drowning, electrocution, and gynecological causes) and 17,839 cases involving pediatric patients (<18 years) or those with missing age data. A further 28,440 cases were excluded where resuscitation was not attempted by EMS personnel or essential clinical data were incomplete. The final study population comprised 147,962 adult OHCA cases where resuscitation was attempted (Figure 1).

### 2.3. Age Stratification and Variable Definitions

Patients were stratified using a 65-year threshold, consistent with geriatric medicine definitions and previous OHCA publication [21]. We analyzed age both as a continuous variable and categorically (elderly: >65 years vs. non-elderly: ≤65 years).

Clinical variables included initial cardiac rhythm (ventricular fibrillation/ventricular tachycardia [VF/VT], pulseless electrical activity [PEA], asystole, bradycardia), witness status (medical personnel, non-medical bystander, unwitnessed), location (home, public, other), and sex. HNAS interventions included shock delivery (automated external defibrillator [AED] or manual defibrillation), mechanical chest compression, and HNAS response times. We distinguished between AED equipment availability and actual shock delivery, as equipment presence does not guarantee its clinical use.

### 2.4. Statistical Analysis

We compared baseline characteristics between elderly and non-elderly patients using chi-square tests for categorical variables and t-tests for continuous variables. Individual associations with survival were assessed using logistic regression, reporting odds ratios (OR) with 95% confidence intervals (CI). We developed a final logistic regression model including clinically relevant predictors. Variable selection was based on clinical importance and statistical significance (*p* < 0.05). Model discrimination was assessed using the area under the receiver operating characteristic curve (AUC). Model calibration was evaluated using the Brier score and calibration plots. Clinical utility was quantified using number needed to treat (NNT) calculations. Analyses were performed using Python 3.8 (Python Software Foundation, Wilmington, DE, USA) with stats models and scikit-learn libraries. Statistical significance was set at *p* < 0.05.

### 2.5. Age-Specific Treatment Effectiveness

We specifically tested whether treatment effects differed by age by including age × treatment interaction terms in the model. We tested interactions for: (1) sex and age, (2) initial rhythm and age, and (3) shock delivery and age. Only interactions with *p* < 0.05 after multiple testing correction are reported.

For each significant interaction, we calculated age-stratified effect sizes and confidence intervals. We report both the interaction odds ratio (how much the treatment effect changes with age) and the clinical interpretation (treatment effectiveness in elderly vs. non-elderly patients).

## 3. Results

### 3.1. Study Population and Baseline Characteristics

During the study period, 147,962 adult out-of-hospital cardiac arrest patients with attempted resuscitation were included in the analysis. The overall survival to hospital discharge rate was 8.8% (n = 12,975). Patients ranged in age from 18 to 107 years (mean: 69.6 ± 14.4 years), with 99,576 (67.3%) classified as elderly (>65 years).

Table 1 presents baseline characteristics stratified by age group. In the elderly population, male sex was more common than female (52.2% vs. 47.8%, *p* < 0.001), they suffered cardiac arrest at home, and more likely presented with non-shockable rhythms. The prevalence of ventricular fibrillation/ventricular tachycardia (VF/VT) was significantly lower in elderly patients (10.6% overall with predominance in younger patients), while asystole was more common for the elderly (70.8% overall, *p* < 0.001). Emergency medical service response times and AED equipment availability were similar between groups.

### 3.2. Age-Stratified Survival Outcomes

**Figure 2:**
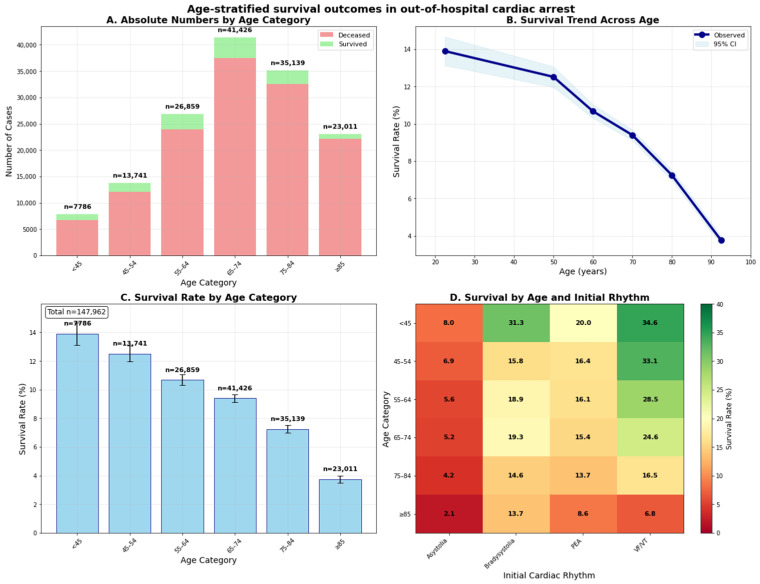
Age-stratified survival outcomes in out-of-hospital cardiac arrest. Comprehensive visualization of patient outcomes and survival patterns across age groups. (**A**) displays absolute patient counts and outcomes by age category, illustrating the increasing burden of mortality with advancing age. (**B**) shows the continuous relationship between age and survival probability with 95% confidence intervals, confirming a progressive decline in outcomes. (**C**) presents survival rates by age group with confidence intervals, highlighting consistent disadvantage among elderly patients. (**D**) depicts survival rates stratified simultaneously by age group and initial cardiac rhythm, demonstrating that survival reductions with age persist across rhythm subtypes, including VF/VT, PEA, and asystole. Together, these analyses underscore a clinically significant age-related survival disadvantage, with implications for tailoring resuscitation strategies and prioritizing elderly specific emergency protocols. CI = confidence interval; PEA = pulseless electrical activity; VF/VT = ventricular fibrillation/ventricular tachycardia.

The primary analysis revealed a pronounced age–survival relationship with significant clinical implications for geriatric emergency medicine. Figure 2 demonstrates the comprehensive age-stratified survival pattern across multiple analytical perspectives: overall survival decreased progressively with advancing age, demonstrating a nearly linear decline from younger to older age groups.

The clinically relevant 65-year threshold revealed a substantial survival disparity: elderly patients (≥65 years) achieved 7.3% survival compared to 11.7% in non-elderly patients (<65 years). This survival disadvantage, concerning any sort of initial cardiac rhythms and clinical presentations, establishes age as a fundamental prognostic factor independent of arrest characteristics.

### 3.3. Individual Predictors of Survival: Univariate Analysis

Prior to multivariate adjustment, univariate analysis identified significant associations between survival and key clinical variables (Table 2). The strongest individual predictors included shockable cardiac rhythms demonstrating the highest survival association compared to asystole (reference category). Witness status by medical personnel conferred substantial survival benefit, while advanced interventions showed varying effects.

AED shock delivery demonstrated remarkable individual impact, reflecting both patient selection (shockable rhythms) and therapeutic effectiveness.

Each additional year of age decreased survival odds by 2%, while notable variations emerged across clinical presentations and emergency response characteristics.

### 3.4. Multivariate Model: Independent Predictors of Survival

**Figure 3:**
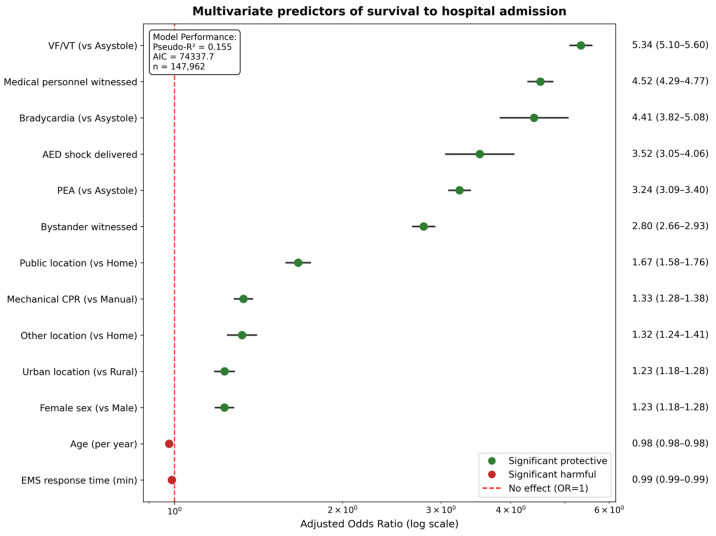
Multivariate predictors of survival to hospital admission. Forest plot displaying adjusted odds ratios and 95% confidence intervals for independent predictors of survival in the final multivariate logistic regression model. Variables are ordered by effect magnitude, with protective factors (OR > 1, green points) and risk factors (OR < 1, red points) clearly distinguished. The model demonstrates strong discrimination and includes clinically relevant variables with clear temporal relationships to outcome. Advanced airway management was excluded due to confounding by indication. The analysis confirms VF/VT rhythm, medical witness, and AED shock delivery as the strongest independent predictors, while demonstrating persistent age-related survival disadvantage even after adjustment for clinical factors. Results support evidence-based emergency protocols while identifying opportunities for targeted interventions. AED = automated external defibrillator; CPR = cardiopulmonary resuscitation; EMS = emergency medical services; OR = odds ratio; PEA = pulseless electrical activity; VF/VT = ventricular fibrillation/ventricular tachycardia.

After adjustment for confounding variables, the final multivariate model included 14 clinically relevant predictors and demonstrated good discrimination (AUC = 0.784, 95% CI: 0.0780–0.788) with good calibration (Brier score = 0.0705). Figure 3 presents the forest plot visualization of adjusted odds ratios, while Table 3 provides detailed effect estimates.

The adjusted analysis confirmed VF/VT rhythm as the strongest independent predictor (adjusted OR 5.34, 95% CI: 5.10–5.60), followed by witness by medical personnel (OR 4.52, 95% CI: 4.29–4.77), bradycardia (OR 4.41, 95% CI: 3.82–5.08) and AED shock delivery (OR 3.52, 95% CI: 3.05–4.06). Age remained a significant independent predictor, with each additional year associated with 2% decreased survival odds (OR 0.98, 95% CI: 0.98–0.98).

### 3.5. Age Interaction Analysis: Treatment Effect Modification

**Figure 4:**
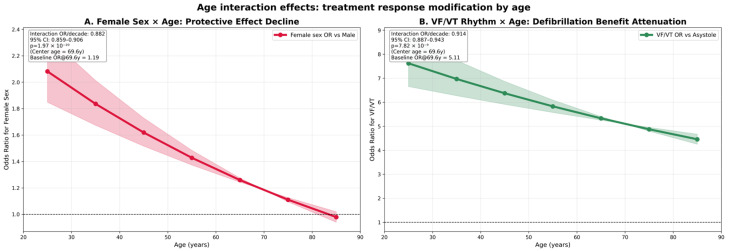
Age interaction effects: treatment response modification by age. Comprehensive visualization of significant age–treatment interactions demonstrates how clinical intervention effectiveness varies across the age spectrum. (**A**) shows the protective effect of female sex progressively declining with advancing age, with odds ratios decreasing from a clear survival advantage in younger patients to near-null effect in elderly patients. (**B**) illustrates the attenuation of the VF/VT rhythm survival benefit with age, though defibrillation remains the strongest independent predictor across all age groups. These findings highlight clinically relevant effect modification, suggesting that demographic and rhythm-related predictors exert differential impacts across the lifespan. Results are derived from multivariable logistic regression models with continuous age interaction terms, centered at the mean population age (69.6 years) and scaled per decade.

A critical novel finding emerged from systematic testing of age-treatment interactions using advanced statistical methodology. After correction for multiple testing using the false discovery rate (FDR) method, three significant interactions demonstrated that treatment effectiveness varies substantially by patient age (Table 4 and Figure 4).

The most clinically relevant interaction involved female sex and age (interaction OR 0.882, 95% CI: 0.859–0.906, *p* < 0.001 after FDR correction).

The VF/VT rhythm–age interaction (OR 0.914, 95% CI: 0.887–0.0943, *p* < 0.001 after FDR correction) revealed that while shockable rhythms remained the strongest predictor across all age groups, the survival benefit was attenuated in elderly patients.

### 3.6. Clinical Impact and Model Performance

The final model demonstrated robust predictive performance with clinical utility across probability thresholds. Table 5 presents comprehensive performance metrics demonstrating good discrimination and calibration. Five-fold cross-validation confirmed model stability, indicating good generalizability without overfitting.

Clinical impact analysis revealed substantial treatment effects quantified through number needed to treat (NNT) calculations. AED shock delivery demonstrated remarkable effectiveness (NNT = 3). A medical personnel witness showed strong impact (NNT = 10), while having a non-medical bystander witness remained clinically beneficial (NNT = 26).

The age-stratified analysis revealed that while overall survival decreased with age, the relative effectiveness of key interventions remained substantial across age groups.

## 4. Discussion

This nationwide Hungarian registry-based analysis provides one of the largest and most detailed evaluations of age-specific survival patterns, independent predictors, and treatment–age interactions in out-of-hospital cardiac arrest (OHCA) to date. The analysis of 147,962 adult OHCA cases confirms a strong and progressive association between increasing age and reduced survival, with each additional year lowering survival odds by approximately 2%. This decline was consistent across the age spectrum and reflects the multifactorial vulnerability of elderly patients. While age independently predicts survival, our results show that treatment effectiveness also varies by age in clinically meaningful ways.

The markedly lower prevalence of VF/VT among elderly patients, alongside the predominance of asystole, has direct clinical implications. VF/VT carried the strongest survival association in our adjusted models, yet its reduced incidence in older patients inherently limits the proportion who can benefit from defibrillation. This finding underscores the importance of early recognition, rapid EMS activation, and targeted preventive measures—such as remote monitoring technologies—in older populations, where delays and comorbidities may allow initially shockable rhythms to deteriorate into non-shockable states or even to initiate cardiac arrest with PEA.

Female sex was independently associated with improved survival overall; however, this benefit diminished with advancing age, indicating that demographic predictors should be interpreted in an age-specific context.

Despite these attenuations, several interventions retained high absolute benefit across all age groups. AED shock delivery had the lowest number needed to treat (NNT = 3), with negligible difference between elderly (3.2) and non-elderly (2.8) patients. This finding supports maintaining universal public-access defibrillation programs emphasizing no age limitations. Similarly, presence of a medical person as a witness has a substantial survival benefit (NNT = 10), while a non-medical bystander witness still produced a meaningful improvement (NNT = 26). These results highlight the importance of rapid intervention at all stages of the chain of survival, regardless of patient age.

Our identification of significant age–treatment interactions provides a basis for developing geriatric-tailored resuscitation approaches, while confirming that core interventions such as defibrillation remain highly effective across the age spectrum. The high performance of our predictive model supports its potential integration into clinical decision-support systems, enabling real-time, age-adjusted prognostication.

### 4.1. Ethical and Implementation Implications

From an ethical and systems perspective, our findings support the European Resuscitation Council position that chronological age alone should not determine resuscitation intensity, because older patients in our cohort still derived meaningful benefit from core evidence-based interventions (notably early defibrillation). At the same time, real-world practice may deviate from this principle: in a case–control analysis, older OHCA patients received shorter and less intensive out-of-hospital care, suggesting that age-related treatment attenuation may contribute to poorer outcomes [10]. These considerations underscore the need for transparent, protocol-driven decision-making and quality-assurance processes that explicitly guard against age-based therapeutic nihilism.

Implementation efforts should also account for where many elderly arrests occur—at home and within municipal/home-care contexts. Qualitative evidence indicates that a lack of organizational support, limited care alternatives, and insufficiently structured collaboration between municipal care personnel and EMS can jeopardize outcomes and reduce older persons’ ability to influence care during emergencies; conversely, care personnel acting as the patient’s representative can strengthen patient agency and facilitate effective EMS engagement [22].

In practical terms, this supports strengthening the “first links” of the chain of survival in older populations through:

(i) targeted AED placement and rapid AED access in high-risk elderly communities;
(ii) tailored training and clear role definitions for home-care/municipal staff to accelerate recognition, calling, CPR/AED initiation, and handover to EMS; and;
(iii) audit/feedback focusing on equitable delivery of time-critical interventions across age strata.

### 4.2. Limitation

This study has several limitations inherent to its retrospective, registry-based design. First, the primary endpoint was survival to hospital admission; while this avoids confounding by in-hospital variations in care, it does not capture long-term survival or neurological status, which are critical patient-centered outcomes. Finally, our multivariate analysis was limited to variables reliably recorded in the HNAS database. Information regarding patient comorbidities and pre-arrest functional status was not systematically available in the EMS registry. Additionally, we excluded certain intra-arrest interventions (such as drug administration) from the primary prediction model to avoid resuscitation time bias, as these interventions are often proxies for longer resuscitation efforts rather than independent predictors of outcomes in the early phase. System-level disruptions (e.g., pandemic effects) may have influenced case-mix and outcomes.

### 4.3. Future Perspectives

Future work should link EMS records to in-hospital and long-term follow up to evaluate neurologically intact survival and functional outcomes and to better partition prehospital versus in-hospital contributors to age-related differences. Enriching registries with frailty/functional measures may improve age-stratified risk estimation and reduce unmeasured confounding. Before clinical implementation, the prediction model should undergo external validation and recalibration in independent EMS systems, consistent with evidence that performance and calibration can vary substantially across cohorts.

## 5. Conclusions

In this nationwide EMS registry of 147,962 adult OHCAs, increasing age independently and progressively reduced survival to hospital admission. However, key time-critical interventions—especially early defibrillation—remained strongly beneficial across all ages, supporting resuscitation decisions based on clinical factors rather than age alone. Age-related effect modification highlights the need for geriatric-informed systems focused on rapid recognition and early shock delivery. Based on the performance of the prediction model, a real-time, age-adjusted risk stratification tool could yield promising result after external validation.

## Figures and Tables

**Table 1:** Baseline characteristics and clinical variables by survival status.

Characteristic	Overall (N = 147,962)	Non-Elderly < 65 (N = 48,386)	Elderly ≥ 65 (N = 99,576)	*p*-Value
**Demographics**				
Survived to admission, n (%)	12,975 (8.8%)	5669 (11.7%)	7306 (7.3%)	<0.001
Age, mean ± SD (years)	69.6 ± 14.4	53.4 ± 9.6	77.5 ± 8.4	<0.001
Male sex, n (%)	86,858 (58.7%)	34,850 (72.0%)	52,008 (52.2%)	<0.001
**Initial Cardiac Rhythm**				
VF/VT, n (%)	17,576 (11.9%)	7003 (14.5%)	10,573 (10.6%)	<0.001
PEA, n (%)	25,260 (17.1%)	7867 (16.3%)	17,393 (17.5%)	<0.001
Asystole, n (%)	103,590 (70.0%)	33,043 (68.3%)	70,547 (70.8%)	<0.001
Bradycardia, n (%)	1536 (1.0%)	473 (1.0%)	1063 (1.1%)	<0.001
**AED Equipment and Usage**				
AED equipment available, n (%)	118,067 (79.8%)	38,615 (79.8%)	79,452 (79.8%)	0.943
AED rhythm analyzed, n (%)	3339 (2.3%)	1281 (2.6%)	2058 (2.1%)	<0.001
Shock delivered, n (%)	972 (0.7%)	474 (1.0%)	498 (0.5%)	<0.001

**Table 2:** Univariate predictors of survival to hospital admission.

Variable	OR	95% CI	*p*-Value	Clinical Context
Age (per year)	0.98	0.979–0.981	<0.001	2% survival decrease per one year increase
Female sex (vs. Male)	1.21	1.16–1.26	<0.001	Protective effect across ages
VF/VT (vs. Asystole)	4.18	4.01–4.36	<0.001	Strongest individual predictor
PEA (vs. Asystole)	2.31	2.17–2.46	<0.001	Intermediate prognosis
Bradycardia (vs. Asystole)	2.22	1.95–2.54	<0.001	Better than asystole
Medical personnel witness (vs. None)	2.74	2.63–2.85	<0.001	Professional early intervention
Non-medical bystander witness (vs. None)	1.55	1.47–1.63	<0.001	Early recognition and CPR
AED shock delivered (vs. No shock)	9.9	8.72–11.24	<0.001	Combined selection and treatment effect
Urban location (vs. Rural)	1.33	1.26–1.40	<0.001	Healthcare access advantage
Public location (vs. Home)	2.12	2.02–2.22	<0.001	Rapid recognition and EMS access
EMS response time (per min)	0.99	0.990–0.991	<0.001	Time-dependent morbidity

**Table 3:** Multivariate model: independent predictors of survival to hospital admission.

Variable	Adjusted OR	95% CI	*p*-Value	Clinical Interpretation
**Initial Cardiac Rhythm (vs. Asystole)**				
VF/VT (vs. Asystole)	5.34	5.10–5.60	<0.001	Strongest predictor—shockable rhythm
Bradycardia (vs. Asystole)	4.41	3.82–5.08	<0.001	Intermediate prognosis rhythm
PEA (vs. Asystole)	3.24	3.09–3.40	<0.001	Better than asystole, worse than VF/VT
**Witness and Early Intervention**				
Medical personnel witness	4.52	4.29–4.77	<0.001	Professional early intervention
AED shock delivered	3.52	3.05–4.06	<0.001	Definitive defibrillation therapy
Non-medical bystander witness	2.8	2.66–2.93	<0.001	Early recognition and CPR/BLS
**Location and Demographics**				
Urban location (vs. Rural)	1.23	1.18–1.28	<0.001	Healthcare access advantage
Public location (vs. Home)	1.67	1.58–1.76	<0.001	Rapid recognition and EMS access
Other location (vs. Home)	1.32	1.24–1.41	<0.001	Non-home environment advantage
Female sex (vs. Male)	1.23	1.18–1.28	<0.001	Persistent protective effect
Mechanical CPR (vs. Manual)	1.33	1.28–1.38	<0.001	Modest benefit for prolonged cases
**Time-Dependent Factors**				
EMS response time (per min)	0.99	0.99–0.99	<0.001	Time-dependent mortality
**Risk Factors**				
Age (per year)	0.98	0.98–0.98	<0.001	Progressive survival decline

**Table 4:** Age interaction analysis: treatment effect modification by age.

Interaction	Interaction OR	95% CI	*p*-Value	FDR-Corrected *p*	Clinical Interpretation
Female Sex × Age	0.882	0.859–0.906	<0.001	<0.001	Protective effect of female sex diminishes with advancing age
VF/VT Rhythm × Age	0.914	0.887–0.943	<0.001	<0.001	Defibrillation survival benefit attenuated with increasing age

**Table 5:** Model performance and population impact metrics.

Performance Metric	Value	95% CI	Clinical Interpretation
**Discrimination**			
AUC	0.784	0.780–0.788	Overall discrimination
Cross-validation AUC	0.784	0.781–0.787	5-fold stratified CV
**Calibration**			
Brier Score	0.0705	0.0702–0.0709	Overall calibration (lower is better)
Hosmer–Lemeshow *p*-value	0.082		Goodness-of-fit (higher suggests no lack of fit)
**Clinical Utility (at optimal threshold)**			
Sensitivity	73.9%	73.2–74.6%	Case detection
Specificity	68.6%	68.4–68.9%	Rule-out performance
PPV	18.4%	18.2–18.7%	Positive predictive value
NPV	96.5%	96.4–96.6%	Negative predictive value
**Population Impact (adjusted ARD & PIF)**			
AED shock delivered—Adjusted ARD (pp)	13.09	13.05–13.13	Adjusted absolute survival gain (percentage points) if universally applied vs. none
AED shock delivered—PIF (universal, %)	147.5	147.0–148.0	Projected relative increase in overall survival with universal exposure
Medical personnel witness —Adjusted ARD (pp)	14.66	14.62–14.70	Adjusted absolute survival gain (percentage points) if universally applied vs. none
Medical personnel witness—PIF (universal, %)	145.2	144.3–146.0	Projected relative increase in overall survival with universal exposure
Non-medical bystander witness—Adjusted ARD (pp)	7.77	7.74–7.80	Adjusted absolute survival gain (percentage points) if universally applied vs. none
Non-medical bystander witness—PIF (universal, %)	61.1	60.7–61.5	Projected relative increase in overall survival with universal exposure

## Data Availability

The raw data supporting the conclusions of this article will be made available by the authors on request.

## Abbreviations

The following abbreviations are used in this manuscript:

AED	Automated external defibrillator
AUC	Area under curve
CI	Confidence interval
EMS	Emergency medical services
EU	European Union
FDR	False discovery rate
HNAS	Hungarian National Ambulance Service
NNT	Number needed to treat
OHCA	Out-of-hospital cardiac arrest
OR	Odds ratio
PEA	Pulseless electrical activity
ROSC	Return of spontaneous circulation
VF/VT	Ventricular fibrillation/ventricular tachycardia
